# Serum proteome‐wide identified ATP citrate lyase as a novel informative diagnostic and prognostic biomarker in pediatric sepsis: A pilot study

**DOI:** 10.1002/iid3.399

**Published:** 2020-12-30

**Authors:** Chunxia Wang, Xiaodong Zhu, Yun Cui, Huijie Miao, Yaya Xu, Xi Xiong, Xiaomeng Tang, Lujing Shao, Yucai Zhang

**Affiliations:** ^1^ Department of Critical Care Medicine, Shanghai Children's Hospital Shanghai Jiao Tong University Shanghai China; ^2^ Institute of Pediatric Critical Care Shanghai Jiao Tong University Shanghai China; ^3^ Department of Pediatric Critical Care Medicine Xinhua Hospital Affiliated to the Medical School of Shanghai Jiao Tong University Shanghai China; ^4^ Clinical Research Unit, Shanghai Children's Hospital Shanghai Jiao Tong University Shanghai China; ^5^ Institute of Pediatric Infection, Immunity, and Critical Care Medicine, Shanghai Children's Hospital Shanghai Jiao Tong University School of Medicine Shanghai China

**Keywords:** ACLY, biomarker, children, diagnosis, immunometabolism, prognosis, sepsis

## Abstract

**Introduction:**

ATP citrate lyase (ACLY) is involved in lipid metabolism and inflammatory response in immune cells. However, the serum level of ACLY and its clinical relevance in sepsis is totally unknown.

**Methods:**

We conducted a prospective pilot study in patients with sepsis admitted to pediatric intensive care unit (PICU) from January 2018 to December 2018.

**Results:**

Higher levels of ACLY were detected in sera of pediatric patients with sepsis than that of healthy children. The area under the receiver operating characteristic curve (AUC) of ACLY for diagnosis of sepsis was 0.855 (95% confidence interval [CI]: 0757–0.952), and an AUC of ACLY for predicting PICU mortality was 0.770 (95% CI: 0.626–0.915). ACLY levels ≤21 ng/ml on PICU admission predicted an unfavorable prognosis among patients with sepsis with a sensitivity of 87.5% and a specificity of 67.6%. Moreover, serum ACLY levels were correlated to platelet count, IL‐18 levels, and monocyte counts in pediatric patients with sepsis, implying the potential roles of ACLY in immunometabolic regulation in sepsis.

**Conclusions:**

ACLY is firstly identified in sera of patients with sepsis. Serum ACLY level is an additional diagnostic and prognostic biomarker in pediatric patients with sepsis.

## Graphical abstract

ACLY is firstly identified in sera of patients with sepsis. Serum ACLY level is an additional diagnostic and prognostic biomarker in pediatric patients with sepsis.The relationship between serum ACLY and IL‐18 and underlying mechanisms involved in pathophysiology of sepsis need further study in the future.

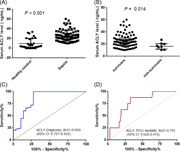

**Abbreviations**
ACLY: ATP citrate lyase
ALT: alanine transaminase
AST: aspartate transaminase
AUC: area under the ROC curve
CRP: C‐reactive protein
ELISA: enzyme‐linked immunosorbent assay
Fib: fibrinogen
IL‐6: interleukin‐6
IL‐10: interleukin‐10
IL‐17A: interleukin‐17A
IL‐18: interleukin‐18
INR: international normalized ratio
Lac: lactate
LPS: lipopolysaccharide
PCT: procalcitonin
PICU: pediatric intensive care unit
PLT: platelet
PT: prothrombin time
ROC: receiver operating characteristic curve
TNF‐α: tumor necrosis factor‐α

## 1 INTRODUCTION

Sepsis is the leading of death in intensive care unit (ICU).^1^ It accounts for 40% of deaths in children younger than 5 years worldwide.^2^ Sepsis is defined as life‐threatening organ dysfunction caused by a dysregulated host response to infection.^3^ The immunopathogenesis of sepsis is a very complex process that involves both overactivation and suppression of immune response.^4^, ^5^ Lymphopenia is a hallmark of sepsis, and profound apoptosis‐induced depletion of lymphocytes including CD4^+^ and CD8^+^ T cells, B cells, and natural killer cells demonstrates extensive loss of lymphocytes in spleens, intestines, and other organs in patients who died of sepsis.^6^, ^7^, ^8^ Recently, Shankar‐Hari et al.^9^ reported that selective apoptotic depletion of memory B cells contributes to the sepsis‐induced immunosuppression. Unfortunately, the evidence about biomarkers for immune status during sepsis is limited, especially in the early stage.

The emergence of the concept of immunometabolism as a major controller of the immune response has raised new hope for re‐recognizing the pathogenesis of sepsis and immunomodulatory therapeutic approaches.^10^ Metabolic reprogramming might reveal to be a novel cornerstone in the treatment of sepsis.^11^, ^12^ Reliable biomarkers could greatly help in accurate assessment of the development and evaluation of new treatments.^13^ The molecules involved in immunometabolism during sepsis could be a potential biomarker for monitoring the immune status, even in predicting the outcome of sepsis.

Up to now, there is no idea about whether the changes of serum protein profiling could reflect the immunometabolic status contributed by activation, secretion, or apoptosis of different immune cells. Our previous study analyzed the changes of serum protein profiling of mice in response to lipopolysaccharide (LPS) stimulation and screened the proteins involved in metabolic molecules.^14^ The mass spectrometry data have been deposited to the ProteomeXchange Consortium via the PRIDE^15^ partner repository with the data set identifier PXD014259. By applying LPS administration in mice, serum ATP citrate lyase (ACLY) was firstly identified (Figure S1). The aim of this study was to assess the value of serum ACLY in patients with sepsis.

## 2 MATERIALS AND METHODS

### 2.1 Patients

Patients with sepsis who were admitted to the pediatric intensive care unit (PICU) at Shanghai Children's Hospital were screened in this study from January 2018 to December 2018. Pediatric sepsis was diagnosed based on the International Pediatric Sepsis Consensus Conference in 2005.^16^ The inclusion criteria included (1) aged with 1 month to 18 years old, (2) diagnosed with sepsis when admitted to PICU, (3) PICU stay over 24 h. The exclusion criteria included (1) advanced tumor or life expectancy <1 month, (2) congenital heart disease, and (3) severe primary diseases or heredity metabolic disease. As a control population, we analyzed 30 residual blood samples from healthy children for a physical examination with normal values for blood counts, C‐reactive protein (CRP), and liver enzymes.

Patients with sepsis were treated in accordance with the International Pediatric Sepsis Consensus Conference in 2005^16^ and Surviving Sepsis Campaign International Guidelines in 2012.^17^ The study protocol was approved by the local ethics committee and conducted in accordance with the ethical standards laid down in the Declaration of Helsinki (Ethics Committee of Children's Hospital affiliated to Shanghai Jiao Tong University [approval number: 2018R039‐F01]). The informed consent was signed by the patients' parents or relatives.

### 2.2 Blood sampling and enzyme‐linked immunosorbent assay (ELISA)

Blood samples were collected at the time point of patient's admission to PICU. Serum ACLY was determined by ELISA (MultiScience [LIANKE] Biotech Co. Ltd.).

### 2.3 Observational variables

According to the pre‐established case report form, we collected the clinical parameters including age, gender, complications, multiple organ dysfunction syndrome (MODS), mean arterial pressure (MAP), systolic blood pressure (SBP), pediatric risk of mortality III score (PRISM III), support of mechanical ventilator or vasoactive agents. The laboratory indexes include lactate (Lac), routine blood indexes (platelet [PLT], the percentage of lymphocyte, monocytes, and neutrophil) and infectious indexes (CRP, procalcitonin [PCT], Lac, interleukin‐8 [IL‐8], IL‐10, IL‐17A, IL‐18, γ‐interferon [γ‐INF], tumor necrosis factor‐α [TNF‐α], IL‐6). The outcome variables including the length of PICU stay and discharged survival status. The laboratory indexes were collected from the first test within 24 h after PICU admission.

### 2.4 Statistical analyses

Data analyses were performed using STATA 15.0 MP. Continuous variables were summarized as means ± *SD* for normal distribution data and as median (interquartile range) for abnormal distribution data. Student's *t* test was used to compare the means of continuous variables and normally distributed data; otherwise, the Mann–Whitney *U* test was used. The *χ* ^2^ test was used to compare the categorical data. Multivariate logistic regression analysis was performed to evaluate the association of serum ACLY with PICU mortality, and the correlation between serum ACLY levels and the proportion of immune cells and inflammatory factors were analyzed. Receiver operating characteristic curve (ROC) was generated to assess the diagnostic or prognostic accuracy in pediatric sepsis. A value of *p* < .05 was considered statistically significant. All *p* values presented are two‐tailed.

## 3 RESULTS

### 3.1 Baseline characteristics of patients in this study

To evaluate the potential value of serum ACLY as a biomarker for sepsis, we measured serum ACLY levels at the time point of PICU admission (before therapeutic interventions) in 101 pediatric patients with sepsis and in 30 healthy controls. These patients included 58 males and 43 females with a median age of 19 (5, 60) months. The baseline characteristics of patients enrolled in this study are shown in Table 1. The PICU mortality rate of pediatric sepsis was 10.9% (11/101), and there were significant differences between survivor and non‐survivor in aspects of PRISM III score (*p* = .007), complications with organ dysfunction including respiratory failure (*p* = .004), shock (*p* = .001), and liver injury (*p* = .004), ratio of mechanical ventilator (*p* < .001) or vasoactive agents supporting (*p* = .001; Table 1).

**Table 1:** Baseline characteristics of patients with sepsis

Characteristics	Healthy control (*n* = 30)	Total (*n* = 101)	Survivor (*n* = 90)	Non‐survivor (*n* = 11)	*p* value
Age (month)	96 (48, 144)	19 (5, 60)	19 (7, 62)	5 (2.5, 45)	.357
Gender (male, %)	12	58	54	4	.134
Emergency, *n* (%)		71	66	5	.056
MAP (mmHg)		86 (75.7, 97.3)	86 (74.6, 96.7)	92 (80, 107)	.208
SBP (mmHg)		101 (88, 112)	100.5 (88, 112)	102 (92, 115)	.563
PRISM III		6 (2, 12)	6 (2, 10)	13 (5, 21)	.007
Complications, *n* (%)					
Respiratory failure		60 (59.4)	49 (54.4)	11 (100)	.004
Shock		45 (44.6)	35 (38.9)	10 (90.9)	.001
Gastrointestinal disorder		35 (34.7)	29 (32.2)	6 (54.5)	.142
Liver dysfunction		11 (10.9)	7 (7.8)	4 (36.4)	.004
Acute kidney injury		17 (16.8)	15 (16.7)	2 (18.2)	.899
Brain injury		31 (30.7)	25 (27.8)	6 (54.5)	.069
MODS		46 (45.5)	35 (38.9)	11 (100)	<.001
Mechanical ventilator, *n* (%)		53 (52.5)	42 (46.7)	11 (100)	<.001
Vasoactive agents, *n* (%)		54 (53.5)	43 (47.8)	11 (100)	.001
Length of PICU stay, day		13 (8, 21)	13 (8, 20)	17 (4, 29)	.711

Abbreviations: MAP, mean arterial pressure; MODS, multiple organ dysfunction syndrome; PICU, pediatric intensive care unit; PRISM III, pediatric risk of mortality III score; SBP, systolic blood pressure.

### 3.2 Serum ACLY level is associated with the occurrence and outcome of sepsis

**Figure 1:**
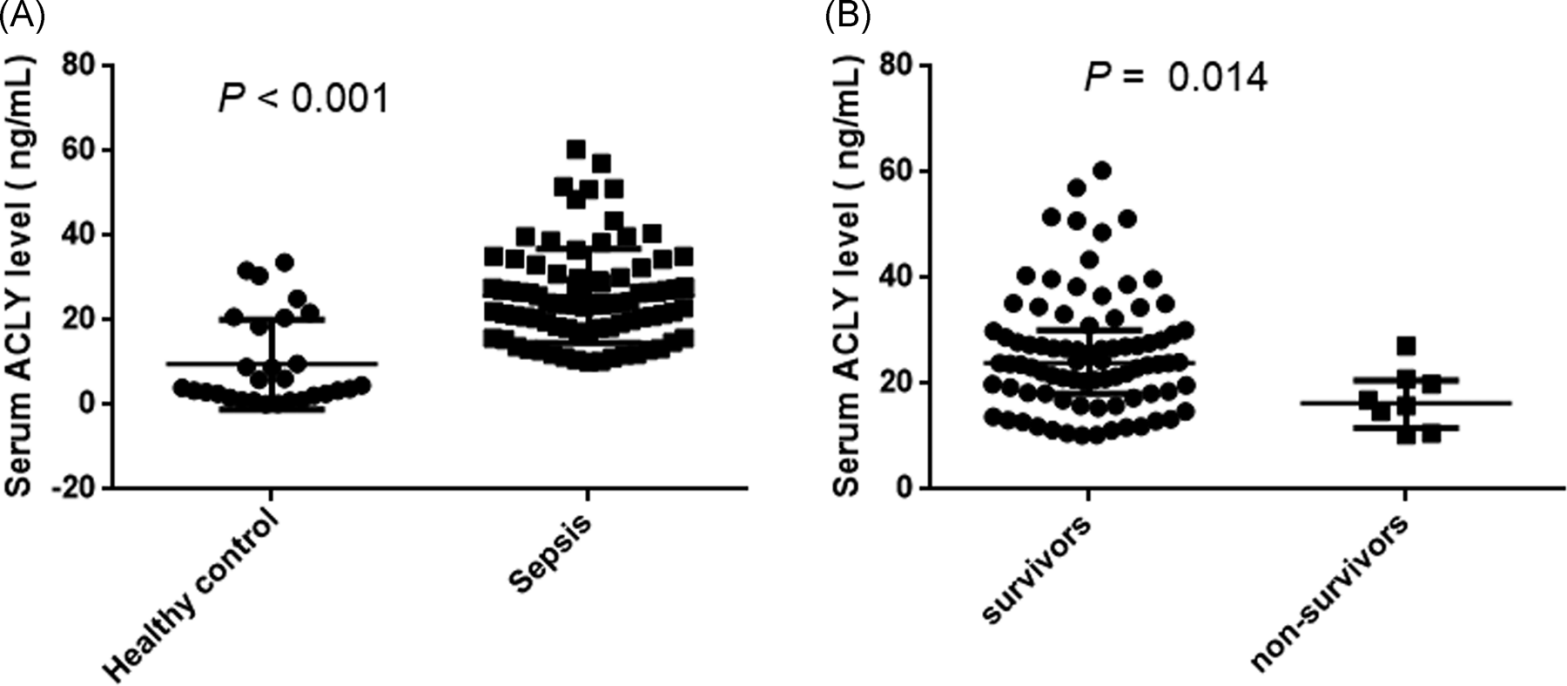
Serum ATP citrate lyase (ACLY) levels in patients with sepsis and healthy control. (A) Healthy control versus sepsis; (B) survivors versus non‐survivors with sepsis

Serum ACLY concentrations on PICU admission were significantly higher in patients who fulfilled sepsis criteria compared with healthy control, and the basic level of serum ACLY in healthy control was very low (Figure 1A). Of note, lower ACLY serum concentration appeared in non‐survivors compared with survivors (Figure 1B). The results of subgroup analysis indicated that there was no significant association between serum ACLY levels and organ dysfunction (all *p* > .05; Figure S2). Moreover, serum ACLY concentrations did not correlate with age or gender, either in controls or in patients (data not shown).

### 3.3 Serum ACLY levels are closely correlated to PLT count, IL‐18 levels, and monocytes count

Besides serum ACLY level, PLT count and the proportion of neutrophils were significantly decreased in non‐survivors compared with the survivors (*p* = .047, *p* = .009, respectively; Table 2). The proportion of lymphocytes was higher in non‐survivors than that in survivors (*p* = .003; Table 2). Among inflammatory factors, levels of IL‐17A and IL‐18 were significantly higher on PICU admission in non‐survivors compared with survivors (*p* = .038, *p* = .027; respectively). The correlation analyses indicated that serum ACLY level was positively correlated to PLT count (*p* = .006) and the levels of IL‐18 (*p* = .027), as well as negatively correlated to monocytes count (*p* = .031) after being adjusted by PRISM III score (Table 3).

**Table 2:** The laboratory indexes of patients with sepsis

Variables	Total (*n* = 101)	Survivor (*n* = 90)	Non‐survivor (*n* = 11)	*p* value
PLT (×10^9^/L)	282 (187, 364)	292.5 (219, 392)	172 (146, 302)	.047
Lymphocyte (%)	21 (10, 31)	18.5 (10, 29)	38 (31, 47)	.003
Neutrophil (%)	72 (58, 83)	73.8 (60.9, 85)	56 (42, 60)	.009
Monocytes (%)	6 (3.4, 9)	6 (3.4, 9)	7 (5, 8)	.683
PCT (ng/ml)	1.22 (0.15, 7.9)	1.19 (0.13, 7.9)	1.82 (0.15, 10.13)	.831
CRP (mg/L)	21 (5, 81)	20.5 (5, 81)	23 (5, 150)	.638
Lac (mmol/L)	1.1 (0.7, 1.9)	1.1 (0.7, 1.9)	1.5 (0.9, 2.6)	.367
Lac‐72 h (mmol/L)	2.1 (1.6, 2.6)	2.0 (1.6, 2.6)	2.7 (1.7, 3.3)	.108
IL‐8 (pg/ml)	1.68 (0.1, 36.93)	0.985 (0.1, 13.52)	47.99 (0.1, 68.96)	.326
IL‐10 (pg/ml)	4.54 (0.1, 19.3)	3.46 (0.1, 13.95)	17.82 (9.73, 44.78)	.054
IL‐17A (pg/ml)	0.1 (0.1, 0.75)	0.1 (0.1, 0.65)	0.91 (0.23, 4.89)	.038
IL‐18 (pg/ml)	95.15 (31.63, 369.44)	60.755 (24.09, 356.04)	479.66 (345.86, 567.02)	.027
γ‐INF (pg/ml)	1.63 (0.29, 5.63)	1.495 (0.29, 5.36)	1.99 (0.98, 9.74)	.795
TNF‐α (pg/ml)	0.1 (0.1, 1.5)	0.1 (0.1, 1.4)	1.0 (0.1, 31.5)	.273
IL‐6 (pg/ml)	45.0 (0.1, 288)	40.9 (0.1, 253.2)	81.5 (48, 14,058.5)	.194

Abbreviations: CRP, C‐reactive protein; IL‐6, interleukin‐6; IL‐8, interleukin‐8; IL‐10, interleukin‐10; IL‐17A, interleukin‐17A; IL‐18, interleukin‐18; Lac, lactate; PLT, platelet; PCT, procalcitonin; TNF‐α, tumor necrosis factor‐α; γ‐INF, γ‐interferon.

**Table 3:** Correlation of serum ACLY Levels to immune cells

Serum ACLY	Coef.	*SE*	*p*	95% CI
Model 1 for immune cells				
PLT	0.018	0.007	0.015	0.004 to 0.032
Monocytes	−0.664	0.285	0.022	−1.231 to −0.096
Lymphocyte	−0.071	0.074	0.342	−0.218 to 0.076
Neutrophil	0.097	0.064	0.134	−0.030 to 0.223
Model 2 for inflammatory factors				
IL‐17A	−0.638	0.340	0.071	−1.336 to 0.059
IL‐18	0.004	0.002	0.030	0.0004 to 0.007
Model adjusted by PRISM III				
PLT	0.020	0.007	0.006	0.006 to 0.035
Monocytes	−0.624	0.284	0.031	−1.189 to −0.059
IL‐18	0.004	0.002	0.027	0.0005 to 0.007

Abbreviations: ACLY, ATP citrate lyase; CI, confidence interval; IL‐17A, interleukin‐17A; IL‐18, interleukin‐18; PLT, platelet; PRISM III, pediatric risk of mortality III score.

### 3.4 Serum ACLY level is an additional diagnostic and prognostic biomarker in pediatric patients with sepsis

**Figure 2:**
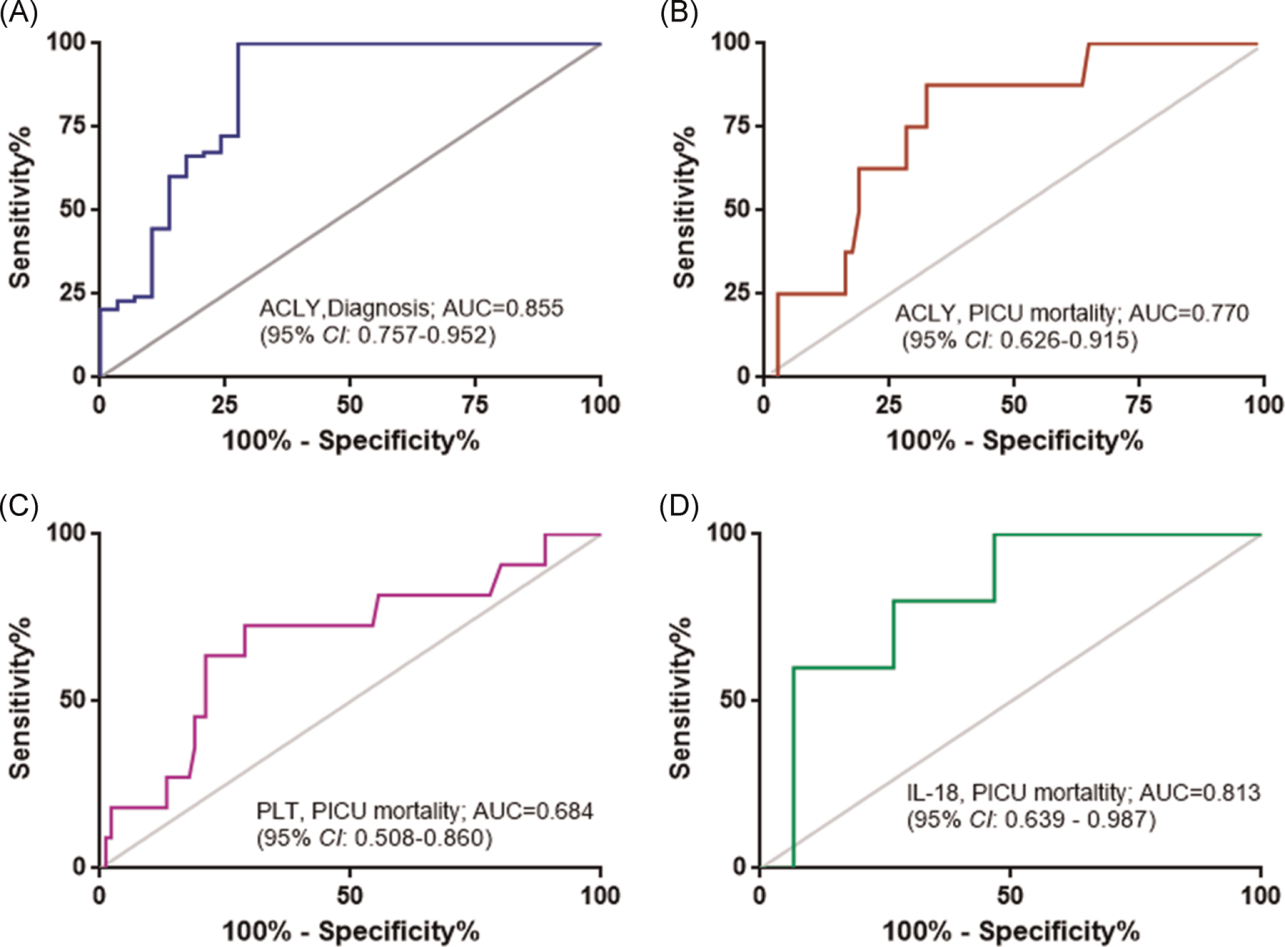
ROC analysis of serum ACLY level for predicting sepsis occurrence and the outcome of pediatric sepsis. (A) ROC analyses of serum ACLY level on PICU admission for predicting sepsis occurrence; (B–D) ROC analyses of (B) serum ACLY, (C) PLT count, and (D) serum IL‐18 level for predicting PICU mortality in pediatric patients with sepsis. ACLY, ATP citrate lyase; AUC, area under ROC curve; CI, confidence interval; IL‐18, interleukin‐18; PLT, platelet; PICU, pediatric intensive care unit; ROC, receiver operating characteristic curve

**Figure 3:**
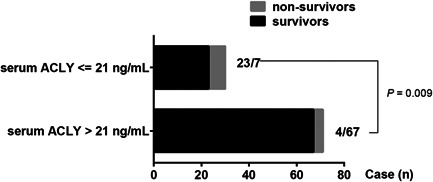
Comparison of the pediatric intensive care unit mortality in the subgroups of septic pediatric patients with serum ATP citrate lyase (ACLY) level ≤21 ng/ml or serum ACLY level > 21 ng/ml

To enclose the potential value of serum ACLY in pediatric sepsis, ROC analysis was performed to assess the power of serum ACLY in assessing the occurrence of sepsis and in predicting the outcome of sepsis. The results showed that serum ACLY levels could be an additional biomarker for sepsis occurrence with a high value of area under ROC curve (AUC) of 0.855 (95% CI: 0.757–0.952; Figure 2A). The cut‐off value of serum ACLY level for an additional diagnosis of sepsis was 9.96 ng/ml with a sensitivity of 100% and a specificity of 72.4%. Furthermore, serum ACLY levels displayed high prognostic accuracy for PICU mortality with an AUC of 0.770 (95% CI: 0.626–0.915), and the sensitivity was 87.5% and the specificity was 67.6% at the cut‐off value of 21 ng/ml (Figure 2B). In addition, the AUC of PLT count or IL‐18 level for predicting PICU mortality was 0.684 (95% CI: 0.508–0.860) or 0.813 (95% CI: 0.639–0.987), respectively (Figure 2C,D). Furthermore, there were seven cases who died in the subgroup of 30 patients with serum ACLY level ≤21 ng/ml, and four cases died in the subgroup of 71 patients with serum ACLY level >21 ng/ml (Figure 3). The PICU mortality was significantly higher in the subgroup of serum ACLY level ≤21 ng/ml compared with the subgroup of serum ACLY level >21 ng/ml (7/30 vs. 4/71; *p* = .009; Figure 3).

## 4 DISCUSSION

To the best of our knowledge, it is the first report about serum ACLY in patients with sepsis. serum ACLY level was correlated to PLT count, IL‐18 levels, and monocytes count, and serum ACLY level proved to be an additional diagnostic biomarker and a prognostic predictor. These results give a new insight into immunometabolic biomarkers to assess the occurrence and outcome of sepsis.

It is well‐known that ACLY is a cytoplasmic enzyme that represents a cross‐link between glucose metabolism and fatty acid synthesis/mevalonate pathways.^18^ Recent studies indicated that ACLY plays a key role in macrophage inflammatory response^19^ and functional capacity in CD8^+^ T cells by translating increased systemic acetate concentrations into increased glycolysis.^20^ Furthermore, increased ACLY expression and enzymatic activity contribute to B‐cell differentiation by mediating glucose‐dependent de novo lipogenesis in response to LPS signaling.^21^ To the best of our knowledge, this is the first study to report the existence of ACLY in the serum of mouse responding to LPS, as well as in pediatric patients with sepsis. ACLY plays a remarkable role in metabolic regulation in cancer cell and would be a promising target for cancer therapy.^22^ There is growing popularity of enclosing the structure and searching the inhibitor of ACLY.^23^, ^24^ Recent studies indicated that ACLY plays a key role in macrophage inflammatory response^19^ and functional capacity in CD8^+^ T cells by translating increased systemic acetate concentrations into increased glycolysis.^20^ The apoptosis of lymphocytes and monocytes might result in releasing the Intracellular molecules to circulation, which could contribute to the appearance of ACLY in serum. In our present study, ACLY was almost undetectable in sera of healthy children, and serum ACLY level achieved a higher diagnostic value for pediatric sepsis. So, it is promising that serum ACLY is a powerful additional predictor for sepsis occurrence.

In this study, we demonstrated a prognostic impact of ACLY serum concentrations on PICU admission in pediatric patients with sepsis. This study first demonstrated that serum ACLY level could be a candidate for the prognostic value of sepsis. In particular, patients with lower ACLY serum levels (≤21 ng/ml) had a significantly impaired prognosis compared with patients with higher ACLY levels (>21 ng/ml). Given the important roles of ACLY in mitochondria function and metabolic regulation,^25^, ^26^ we could further speculate that ACLY may be implemented into established scoring systems to further improve their sensitivity and specificity, and maybe a drug target to regulate immune response via ACLY‐mediating metabolism for sepsis, just like in cancer. However, this assumption is based on a single‐center analysis and future prospective studies are needed to test this hypothesis.

As a novel prognostic predictor, the correlations between serum ACLY and other laboratory indexes give some implications for its function in sepsis. PLTs have received increasing attention for their role in the pathophysiology of sepsis via regulating inflammatory and immune responses, clotting cascade, and endothelial injury.^27^ The relation between PLT parameters and outcome in septic patients has been supported by numerous studies.^28^, ^29^ However, there is a limited study about the role of PLT count on PICU admission in sepsis. Thrombocytopenia is defined as a PLT count <150 × 10^9^/L and the severity of thrombocytopenia positively correlates to the severity of inflammation and the hospital mortality in adult patients.^30^, ^31^ In our study, PLT count on PICU admission is associated with PICU mortality, and PLT count is also positively correlated with serum ACLY level. The underlying mechanisms of PLT count influencing serum ACLY level need further investigation. Otherwise, we found that serum ACLY levels were correlated with serum IL‐8 levels. IL‐18 originally named as “interferon‐c‐inducing factor,” and IL‐18 potentiated mortality in both neonatal sepsis and endotoxemia through the induction of IL‐17A. In addition, targeting IL‐18/IL‐1/IL‐17A axis may improve outcomes for human neonates with sepsis.^32^ Consistently, we found the lower levels of ACLY and the higher levels of IL‐18 in non‐survivors with septic shock than that in survivors. A recent study indicated that elevated plasma IL‐18 level was associated with increased mortality in patients with acute respiratory distress syndrome with a hazard of death of 2.3 (95% CI, 1.7–3.1).^33^ Otherwise, there was no prognostic impact about IL‐18 in adult patients with sepsis, and IL‐18 was a biomarker better differentiating sepsis and septic shock status than PCT, CRP, and WBC.^34^ Furthermore, several studies indicated that IL‐18 enhances immunosuppressive response by promoting differentiation into monocytic myeloid‐derived suppressor cells (MDSCs), by inducing MDSCs migration into the tumor tissue.^35^, ^36^, ^37^ Sepsis‐induced immunosuppression is the main cause for poor outcome of sepsis contributed by metabolic failure, epigenetic reprogramming, MDSCs, and so forth.^38^ So, higher levels of IL‐18 might contribute to immunosuppression in non‐survivors. It is noteworthy that whether the changes of IL‐18 results from ACLY‐mediated metabolic changes in immune cells in the future. In addition, there was a tendency of increased IL‐10 levels in non‐survivors compared with survivors. Given that the correlation between serum levels of ACLY and monocytes count, we suspect that apoptosis of monocytes results in releasing ACLY to serum, which needs further investigation in the future.

Several limitations of this study should be considered. First, the sample size of non‐survivor in this study was relatively small, and it was a single‐center cohort study. Second, the changes of serum levels of ACLY during PICU stay and the correlation of serum ACLY to metabolite levels like citrate or fatty acids were lacking due to the limited blood sampling. Third, how ACLY is released into circulation is still unclear. At present, it is totally unclear which molecular mechanisms are responsible for alterations in serum ACLY levels in sepsis. Moreover, it is still unknown if changes in serum ACLY levels reflect an active secretion to play a functional role as “messengers” or a passive release during cell death. Nevertheless, our findings strongly support future functional and clinical analyses on the role of ACLY in the pathophysiology of sepsis.

In summary, we first identified serum ACLY as a new diagnostic and prognostic biomarker in pediatric patients with sepsis. The relationship between serum ACLY and IL‐18 and underlying mechanisms involved in the pathophysiology of sepsis need further study in the future.

## CONFLICT OF INTERESTS

The authors declare that there are no conflict of interests.

## AUTHOR CONTRIBUTIONS

Chunxia Wang and Yucai Zhang conceived and designed the study and wrote the paper. Chunxia Wang, Huijie Miao, Xiaodong Zhu, Yaya Xu, Xi Xiong, Lujing Shao, and Xiaomeng Tang collected and analyzed data. Xiaodong Zhu, Yun Cui, Chunxia Wang and Yucai Zhang contributed to the analysis of tools and discussion.

## Supporting information

Supporting information.

Supporting information.

Supporting information.

## Data Availability

The data that support the findings of this study are available from the corresponding author upon reasonable request.
