# Supplementary figures and images for: Serum proteome‐wide identified ATP citrate lyase as a novel informative diagnostic and prognostic biomarker in pediatric sepsis: A pilot study
Source: Immun Inflamm Dis. 2020 Dec 30;9(2):389–97. doi: 10.1002/iid3.399 (PMC8127565; doi:10.1002/iid3.399)

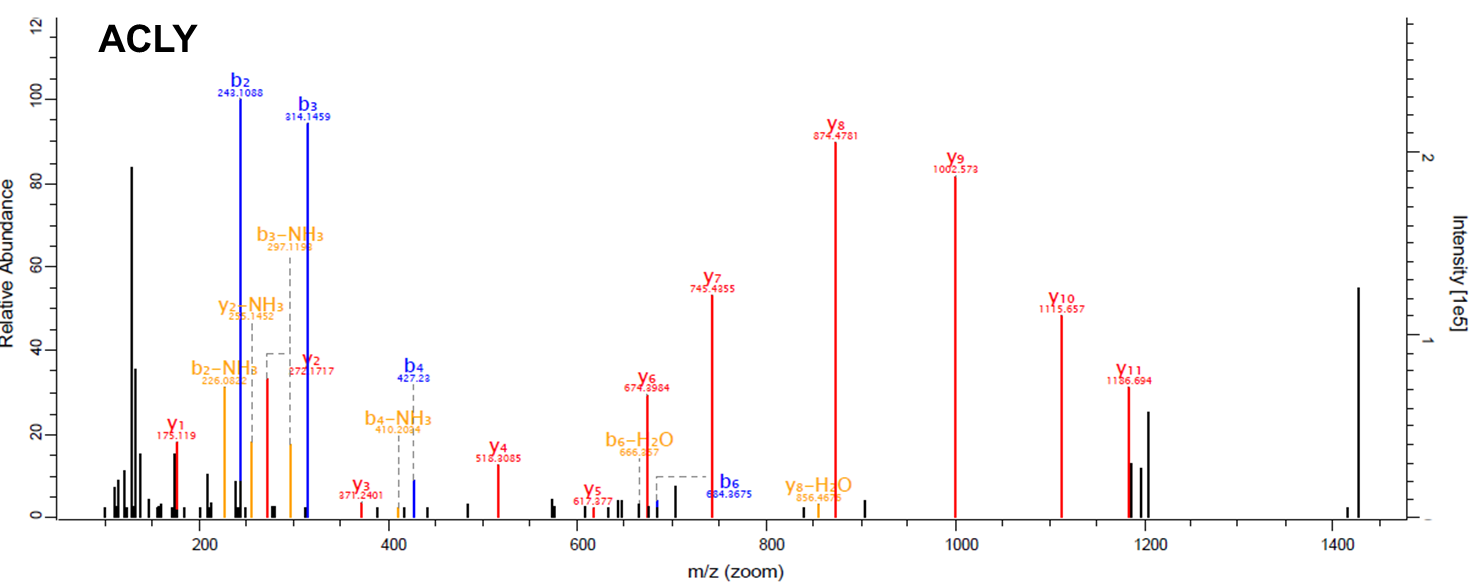

Supplement: Supplementary file 1 — Supporting information. [file IID3-9-389-s003.tiff]

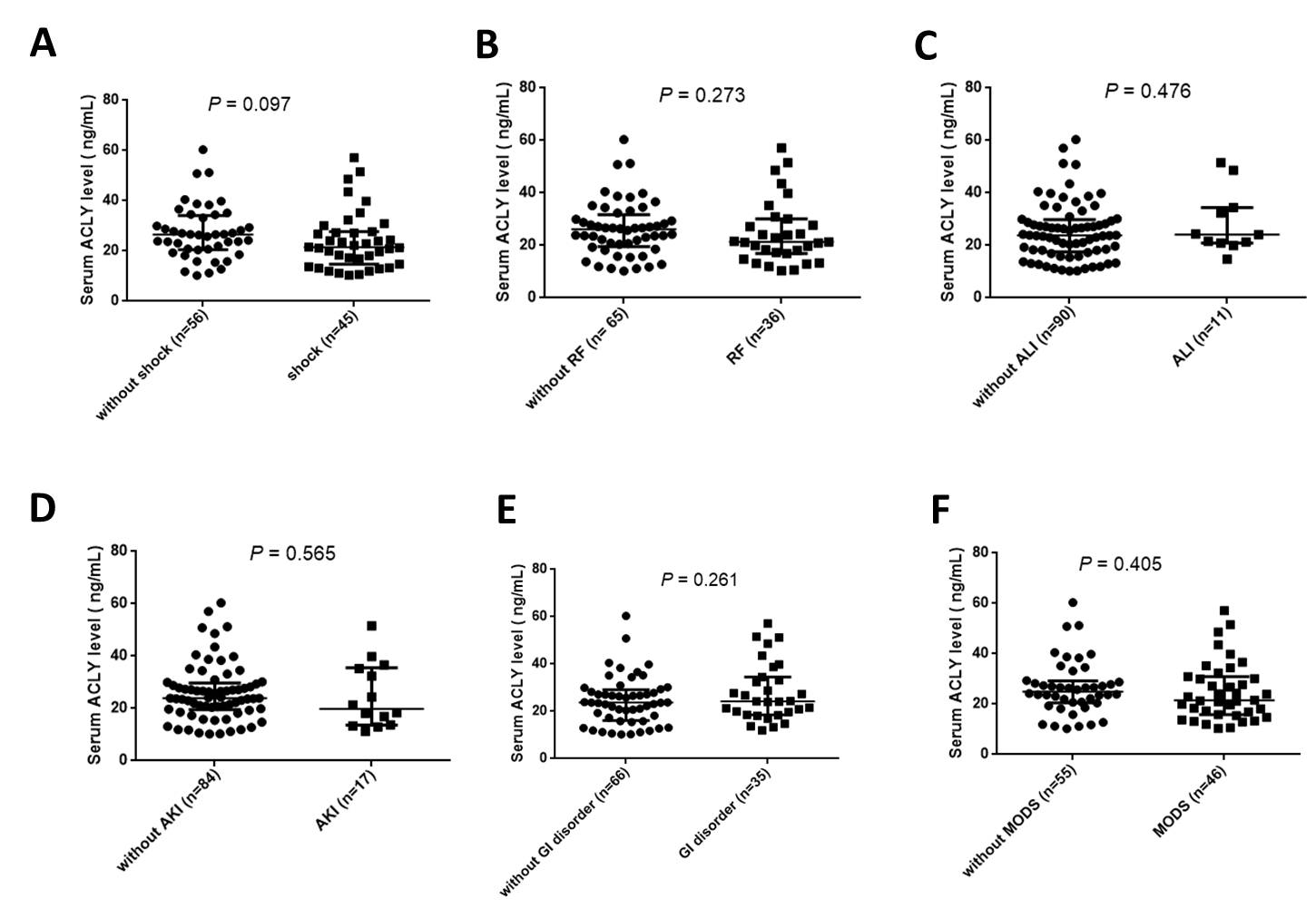

Supplement: Supplementary file 2 — Supporting information. [file IID3-9-389-s001.tiff]
