# Supplementary material for: Serum proteome‐wide identified ATP citrate lyase as a novel informative diagnostic and prognostic biomarker in pediatric sepsis: A pilot study
Source: Immun Inflamm Dis. 2020 Dec 30;9(2):389–97. doi: 10.1002/iid3.399 (PMC8127565; doi:10.1002/iid3.399)
Supplement: Supplementary file 3 — Supporting information. [file IID3-9-389-s002.docx]

**Supplementary Figure 1.** MS/MS spectra used for the identification of ACLY.

**Supplementary Figure 2. Serum ACLY levels in the subgroups of patients with different organ dysfunction**

**(A)** without shock *vs.* shock; **(B)** without respiratory failure (RF) *vs.* RF; **(C)** without acute liver injury (ALI) *vs.* ALI; **(D)** without acute kidney injury (AKI) *vs.* AKI; **(E)** without gastrointestinal (GI) disorder *vs.* GI disorder; **(F)** without MODS vs. MODS.
